# Allele-specific expression is widespread in *Bos indicus* muscle and affects meat quality candidate genes

**DOI:** 10.1038/s41598-020-67089-0

**Published:** 2020-06-23

**Authors:** Marcela Maria de Souza, Adhemar Zerlotini, Marina Ibelli Pereira Rocha, Jennifer Jessica Bruscadin, Wellison Jarles da Silva Diniz, Tainã Figueiredo Cardoso, Aline Silva Mello Cesar, Juliana Afonso, Bruno Gabriel Nascimento Andrade, Mauricio de Alvarenga Mudadu, Fabiana Barichello Mokry, Polyana Cristine Tizioto, Priscila Silva Neubern de Oliveira, Simone Cristina Méo Niciura, Luiz Lehmann Coutinho, Luciana Correia de Almeida Regitano

**Affiliations:** 1 Animal Biotechnology, Embrapa Pecuária Sudeste, São Carlos, SP Brazil; 2 Post-graduate Program of Evolutionary Genetics and Molecular Biology, Federal University of São Carlos, São Carlos, SP Brazil; 3 Bioinformatic Multi-user Laboratory, Embrapa Informática Agropecuária, Campinas, SP Brazil; 4 Department of Animal Science, University of São Paulo, Piracicaba, SP Brazil; 5 NGS Soluções Genômicas, Piracicaba, SP Brazil

**Keywords:** Gene expression, Genomics, Transcriptomics

## Abstract

Differences between the expression of the two alleles of a gene are known as allele-specific expression (ASE), a common event in the transcriptome of mammals. Despite ASE being a source of phenotypic variation, its occurrence and effects on genetic prediction of economically relevant traits are still unexplored in bovines. Furthermore, as ASE events are likely driven by cis-regulatory mutations, scanning them throughout the bovine genome represents a significant step to elucidate the mechanisms underlying gene expression regulation. To address this question in a *Bos indicus* population, we built the ASE profile of the skeletal muscle tissue of 190 Nelore steers, using RNA sequencing data and SNPs genotypes from the Illumina BovineHD BeadChip (770 K bp). After quality control, 820 SNPs showed at least one sample with ASE. These SNPs were widespread among all autosomal chromosomes, being 32.01% found in 3′UTR and 31.41% in coding regions. We observed a considerable variation of ASE profile among individuals, which highlighted the need for biological replicates in ASE studies. Functional analysis revealed that ASE genes play critical biological functions in the development and maintenance of muscle tissue. Additionally, some of these genes were previously reported as associated with beef production and quality traits in livestock, thus indicating a possible source of bias on genomic predictions for these traits.

## Introduction

The mechanisms that regulate gene expression are relevant sources of phenotypic variation among individuals. As an example, modulation of the expression level of specific genes is responsible for phenotypic differences related to tissue differentiation and stages of development in cells with the identical genetic makeup. Specific genomic regions can control the expression of some genes, acting intrinsically or in response to environmental stimuli. These regulatory elements are known as expression quantitative trait loci (eQTL) and can be classified either as *cis,* when they affect the expression of adjacent genes on the same DNA molecule, or as *trans,* when they affect physically distant genes.

As a result of gene expression regulation, unlike expected by the Mendelian inheritance model, alleles can be unequally expressed, *i*.*e*., the abundance of messenger RNA (mRNA) from the alleles encoded in paternally and maternally inherited chromosomes is imbalanced^1^. This event is known as allele-specific expression (ASE). In some cases, only one allele is expressed while the other is silent, a pattern called monoallelic expression. There is much evidence that unbalanced expression of alleles is common throughout the mammalian genome^2,3^, and that it may have effects on phenotypic variation^4–6^.

There are many causes of ASE; however, one of the most common is the presence of polymorphisms at regulatory sites acting in *cis*, as *cis*-eQTLs. These polymorphisms, for instance, may lie on target sites of transcription factors (TFs), compromising the affinity to its binding site, and leading to changes in the transcription rate for the phased allele. When ASE is governed by epigenetic events, for example when DNA methylation is distinct between maternal and paternal inherited chromosomes, the resulting expression pattern is dependent on the parental origin of the allele, an event known as genomic imprinting^7^.

Given that the imbalanced allelic expression usually indicates that a *cis*-regulation of the gene expression is taking place, ASE analysis is used for detecting *cis*-regulatory elements, as *cis*-eQTLs, thus complementing traditional methods of genetic linkage studies^8^. These regulatory elements may encompass the ASE SNP itself or may lie within the gene promoters or enhancers, though it must be located on the same chromosome. Lagarrigue *et al*.^8^ observed that 40% of the *cis*-eQTLs affecting ASE genes were not identified by traditional methods. Khansefid *et al*.^6^ reported an overlapping between ASE and local eQTL underlying complex traits in cattle. However, there is a small similarity between studies applying traditional *cis*-eQTLs and ASE approaches, as reported by Hasin-Brunmshtein *et al*.^9^.

Thereupon, the ASE study is a powerful ally to identify the presence of *cis*-regulatory elements. ASE has been used as a starting point for several studies, such as aseQTL, in which sequence variations correlated with unequal allele expression are identified, complementing the eQTL studies^3,6,8,10^. They are also useful for mapping interaction effects between genotype and environment, known as response expression quantitative trait loci (reQTL)^11^. Furthermore, ASE data can be integrated with DNA methylation, providing insights to the effects of methylation on the allelic expression, supporting imprinted gene discovery and parental origin effect determination^12^.

Humans and mice have extensive literature associating allelic expression patterns with phenotypes, especially in cancer and other diseases^13–17^. However, to the best of our knowledge, only three studies described ASE genome-wide using cattle RNA sequencing data, all with *Bos taurus* breeds and using the former version of the bovine genome (UMD3.1). One study found an extensive variation of ASE in 18 different tissues of a single dairy cow^3^. The second one, used four datasets^6^ to detect ASE and compare with local eQTLs. The four datasets included 45 Angus bulls’ muscle samples, 37 Angus bulls’ liver samples, and 20 Holstein cows’ white blood cells (WBC) and liver samples. The latest study found ASE being frequent in 19 Limousine muscle samples^18^.

Despite the over mentioned studies, there is a giant chasm between the knowledge on allelic expression of cattle and humans, reinforcing the need for the development of new studies for bovine genes. Moreover, allele frequencies and linkage disequilibrium between the ASE and *cis* regulatory variations are population parameters that may not be conserved across samplings.

Describing ASE for genes or QTLs affecting complex traits of economic relevance in animal production might find application in animal breeding programs, contributing to increasing the accuracy of predictive models by taking into account distortions from the additive model.

Here, we present a genome-wide analysis of ASE in the skeletal muscle *Longissimus thoracis* of a large sample of Nelore (*Bos indicus*) steers, describing the variation among individuals and the presence of allelic expression pattern on genes associated with meat quality and other production traits, thus contributing to the development of accurate strategies for including genomic information in animal breeding programs.

## Results

### Genotyping and sequencing data analysis

After applying the genotyping filters, 429,513 SNPs out of 695,888 were kept and used for further analysis. Two animals were removed from the original data set due to high missing genotype rates. The average of sequenced reads was 27 million and approximately 35% of them mapped uniquely to only one allele per sample, ranging from 22.4% to 61.26%. Mapping statistics are described in the Supplementary Table S1. The genotyping data with known linkage phase for each animal and the reads resulting from the RNA-seq alignment to the reference genome (*Bos taurus* ARS-UCD1.2) were used to identify the 1,084 SNPs that attended the criteria of having a minimum of 20x coverage in at least one heterozygote sample, which allowed them to be tested for allele-specific expression, against 76,933 SNPs having less than 20x coverage in the heterozygous samples. Thus, from 78,017 potentially transcribed SNPs in muscle, we tested 0.013%. It means that only 0.001% of the 729,962 SNPs represented in the Illumina BovineHD BeadChip met the minimum criteria (genotyping filters, heterozygous samples, within a transcribed region and RNA-seq mapping coverage) to be tested for ASE in our population.

### Genome-wide ASE profiles in Nelore muscle

**Figure 1:**
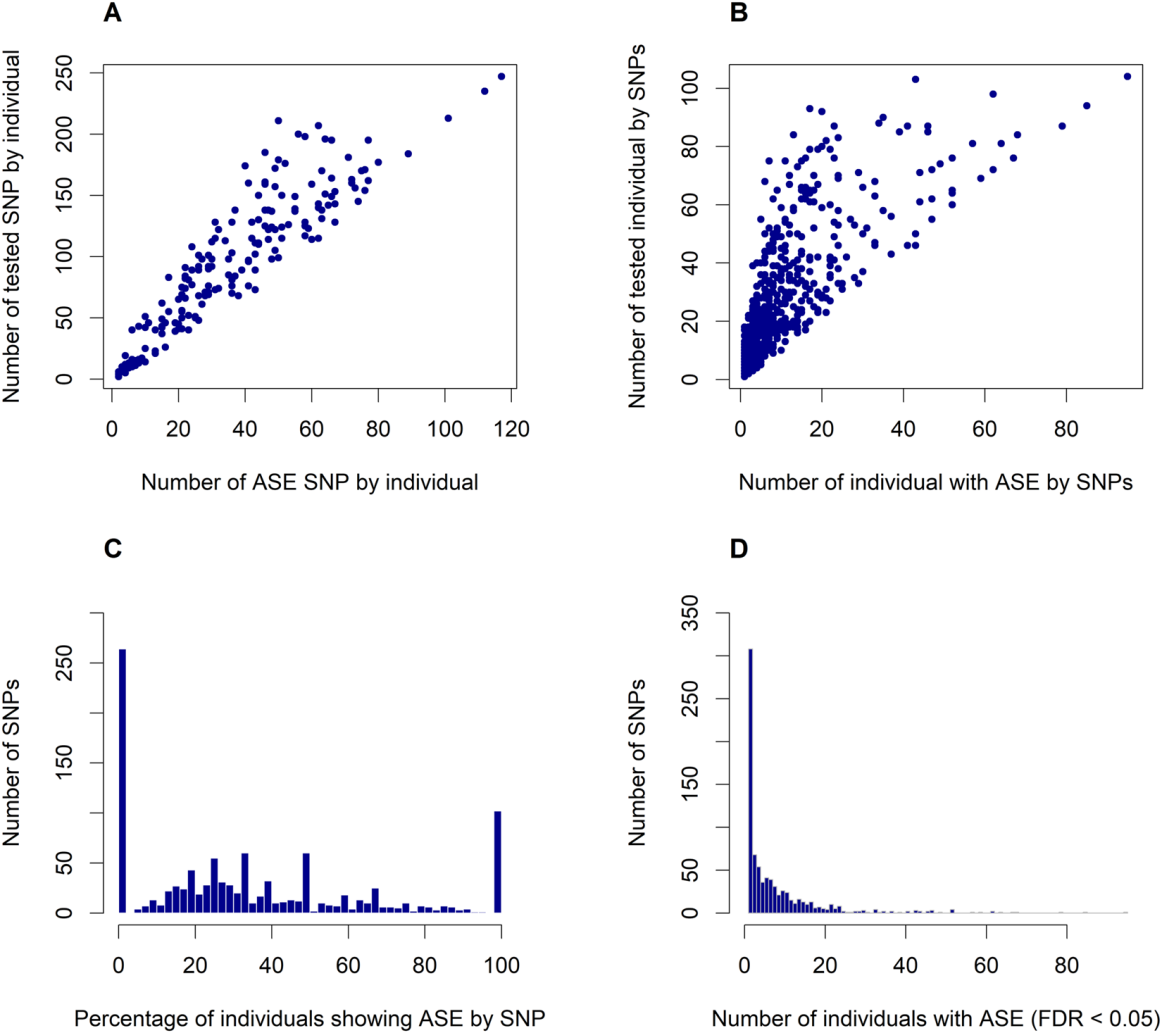
Allele-specific expression (ASE) profile in Nelore. (A) The number of ASE SNPs in function of tested SNPs per individual (R^2^ = 0.95, p = 0.006). Each blue dot represents a individual. **(B)** Number of ASE individuals in function of individuals tested per SNP (R^2^ = 0.74, p = 0.008). Each blue dot represents an SNP. **(C)** Percentage of individuals showing ASE for a given SNP as a proportion of the total individuals tested for the same SNP. **(D)** Distribution of occurrence of ASE through the individuals for each SNP.

In order to identify ASE, each heterozygote SNP locus was tested per individual resulting in approximately 38% significant tests (N = 6,744 out of 17,757) (FDR < 0.05). In summary, from 1,085 SNPs tested, 820 (75.57%) presented ASE in at least one individual, thus being considered as an ASE SNP. Supplementary Table S2 describes the ASE SNPs and Supplementary Table S3 contains the list of SNPs showing biallelic expression. We found a substantial ASE variation among tested individuals (Fig. 1A) and there was a strong correlation between the number of SNPs tested and the number of ASE SNPs per sample (R^2^ = 0.95, p = 0.006; Fig. 1A). The number of SNPs tested for ASE per individual across all 190 samples, ranged from two to 247 (Fig. 1A). Likewise, the number of ASE SNPs per individual ranged from two to 117, with an average of 35.5 (Fig. 1A).

We observed a considerable variation on the number of testable samples and percentage of ASE samples among SNPs (Fig. 1B) which had a positive correlation. As expected, we found the proportion of the individuals with ASE positively correlated with the number of tested individuals by SNP (R^2^ = 0.74, p < 0.008; Fig. 1B). Furthermore, Fig. 1B shows that some SNPs presented consistent ASE throughout the individuals, exhibiting significant results in almost all tested samples, while others seemed to be individual-specific. This also can be observed in Fig. 1C, which exhibits the percentage of tested individuals showing ASE varied among SNPs.

The frequency of significant individuals for a given ASE SNP ranged from one to 95 (Fig. 1B). SNPs with one ASE individual represented approximately 24.51% (N = 201), while 47.56% (N = 390) of the SNPs had ASE in five or more individuals. Only 1.7% (N = 14) of the SNPs showed consistency in more than 50 individuals (Fig. 1D).

When considering the proportion of tested samples per ASE SNP, after excluding SNPs with only one sample tested (N = 71), we found 31 SNPs with all tested individuals showing ASE (100% consistency). In total, 21 SNPs had two testable individuals, eight had between three and five tested individuals, while two SNPs had ten. The complete list of percentages of tested samples for all the 820 ASE SNPs is available in Supplementary Table S2.

From the 265 SNPs showing biallelic expression, *i*.*e*., SNPs with no individuals showing ASE (Supplementary Table S3), rs133595722 and rs109150421 stood out because they had the highest number of tests, 14 and 12, respectively. The remaining SNPs presented between one (56.98%) and ten individuals tested.

When considering SNPs showing low consistency, 120 SNPs had ASE in less than 20% of individuals. Among these SNPs, rs132806881 and rs135381825 showed the smallest proportion of ASE individuals, both having only one ASE out of 18 tested (5.55%).

We also checked if the same allele had higher expression across individuals for a given SNP. For this, we considered only SNPs that had five or more ASE individuals, accounting for 390 SNPs. Approximately 25.89% (N = 101) of the SNPs had higher expression of the same allele in all individuals. Out of that, 84 SNPs had the major allele more expressed, whereas 17 had the minor allele. The remaining 289 SNPs showed both, either the major or the minor allele, as the most expressed among individuals.

### Monoallelic expression

**Figure 2:**
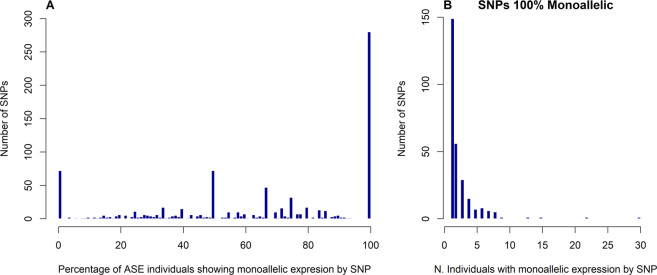
Proportion of monoallelic expression. (**A**) Percentage of ASE individuals showing monoallelic expression for a given SNP as a proportion of the total of individuals with ASE for the same SNP. **(B)** Distribution of the number of monoallelic individuals for SNPs with 100% of monoallelic expression.

The ASE pattern was further analyzed for the individuals who presented monoallelic expression for a given SNP (Fig. 2a). Figure 2b shows that most of the SNPs with 100% of monoallelic expression had only one sample analyzed (N = 149). When we analyzed SNPs with two or more individuals showing ASE (N = 619), we identified 131 SNPs with monoallelic expression for all ASE samples, ranging between two and 30 individuals per SNP (mean 3.91 ± 3.56). In contrast, 20 ASE SNPs did not present any individual with monoallelic expression. Among the remaining SNPs, 314 had monoallelic expression in at least half of the ASE individuals and 154 SNPs had monoallelic pattern in less than 50% of ASE individuals. The SNPs rs41255155 and rs137833130, for which all samples with ASE expressed only one allele, had the highest number of biological replicates within the monoallelic SNPs, with 30 and 22 individuals, respectively. None of the SNPs had 100% of testable individuals showing monoallelic expression. All monoallelic percentages for the ASE SNPs are described in Supplementary Table S2.

### Distribution of ASE

**Figure 3:**
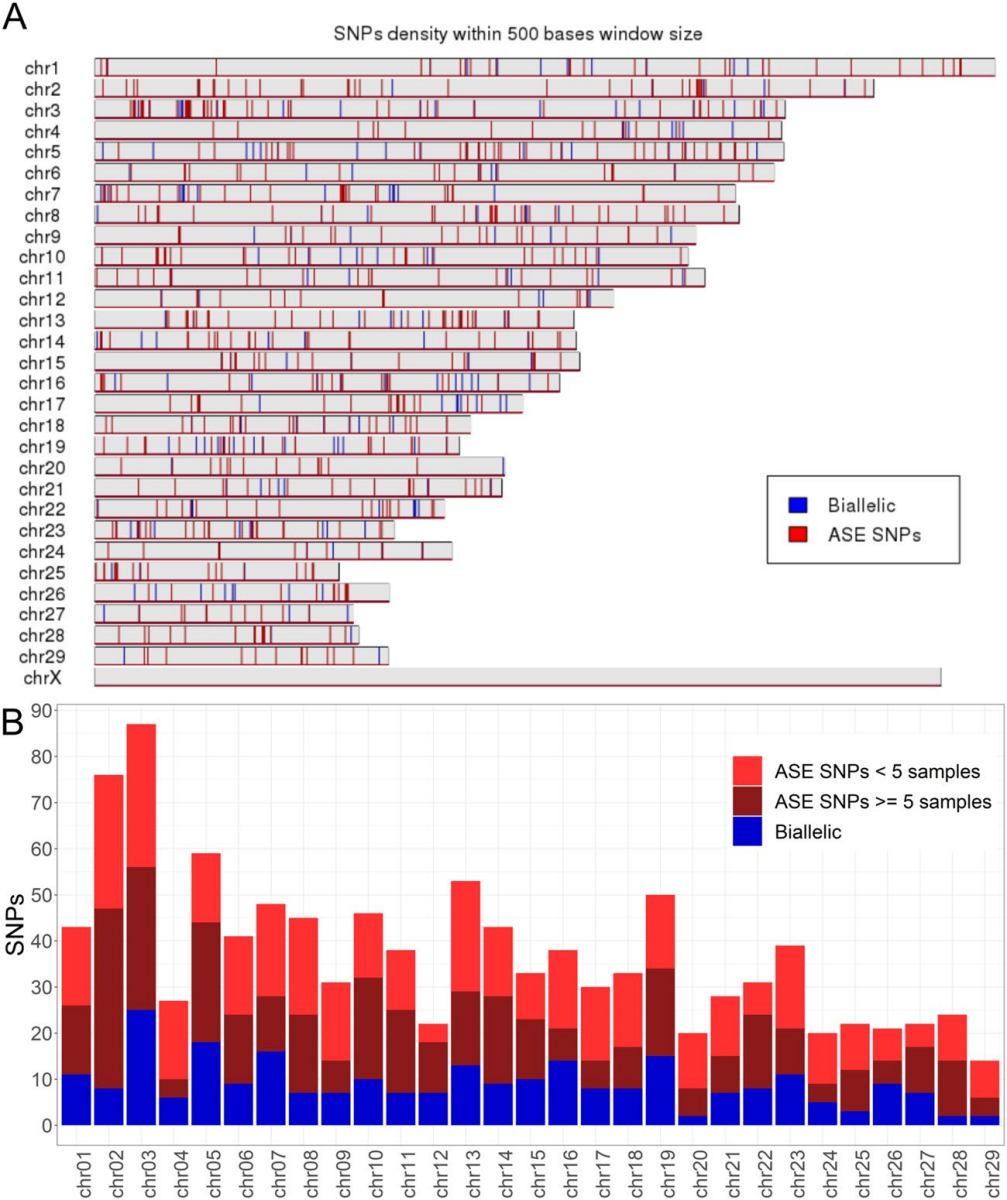
Genomic distribution of ASE SNPs. **(A)** Distribution of all allelic expression through the chromosomes. **(B)** Number of tested SNPs showing or not ASE in two scenarios (less than five samples and five or more samples) for each chromosome. Blue bars represent the SNPs showing biallelic expression in all tested individuals, dark red bars represent SNPs showing ASE in five or more samples (ASE SNPs > = 5 samples), and light red bars represent SNPs showing ASE in less than five individuals (ASE SNPs <5 samples).

The allelic expression was observed to be spread throughout the genome (Fig. 3a) as well as the varying proportions of ASE/biallelic SNPs (Fig. 3b). The proportion of tested SNPs showing ASE per chromosome ranged from 57% (chr 26) to 92% (chr 28), when considering all ASE SNPs, and from 15% (chr 04) to 52% (chr 22) when considering SNPs with at least five ASE samples. It should be noted that the analysis performed here used only transcribed autosomal SNPs represented on the Illumina BovineHD BeadChip (770 K), which means that not all of the muscle expressed bovine genome genes were analyzed.

### Annotation of SNPs and genes showing ASE

**Figure 4:**
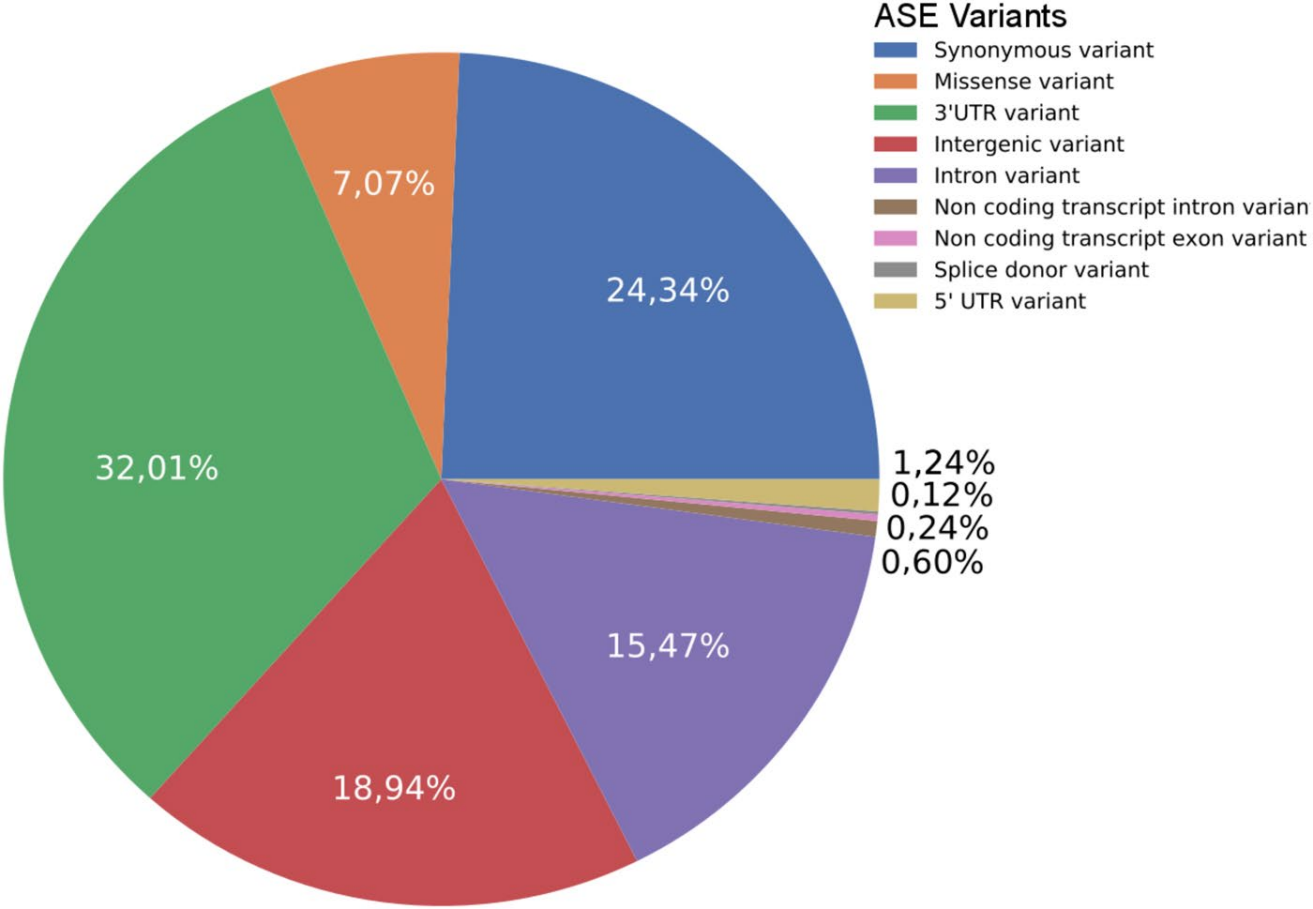
Distribution of ASE variants according to their annotation.

According to the annotation, 32.01% of the ASE SNPs were in 3 ‘UTR regions (Fig. 4). SNPs in coding regions had the second highest representativeness, with 31.41%, from which 7.07% were missense variants, followed by SNPs in intergenic regions and introns, with 18.94% and 15.47%, respectively. The less represented SNP’s positions were 5 ‘UTR (1.20%), and non-coding transcripts or within splicing regions (less than 1%). Close to 23% of the variants located in coding regions may result in amino acid exchange in the corresponding protein.

In total, 820 SNPs located within 530 different genes exhibited at least one individual with ASE, some genes with more than one ASE SNP. Table 1 displays the top 10 genes showing higher consistency featured as higher number of ASE individuals and higher proportion of ASE/tested SNPs. Altogether, 56 genes enclosed two different SNPs each; 11 genes had three SNPs, three genes had four SNPs, and four had five SNPs. The genes *MYL1*, *ARSG* and *CMYA5* had six, seven, and eight SNPs, respectively. Also, 12 SNPs overlapped in two different genes and the SNP rs109895497 overlapped three different genes (Table S2).

**Table 1:** Top 10 genes/SNPs with higher consistency of ASE. Higher consistency was the combination of the highest number of individuals presenting allele-specific expression (ASE) and higher proportion of significant ASE within tested individuals.

Gene Symbol	SNP	ASE individuals (P < 0.05)	Total tested	ASE proportion (%)	Gene location
*CMYA5*	rs133758443	43	46	93.48	synonymous
*CASQ1*	rs109202994	79	87	90.80	3′ UTR
*TXNIP*	rs110233071	85	94	90.43	3′ UTR
*SPARC*	rs137157041	41	46	89.13	3′ UTR
*PDE4DIP*	rs134710361	67	76	88.16	synonymous
*HSPA1A*	rs110850310	52	60	86.67	3′ UTR
*DNAJC21*	rs135102452	37	43	86.05	synonymous
*CMYA5*	rs134932738	43	50	86.00	missense
*TXNIP*	rs41257141	68	84	80.95	5′ UTR
*PDLIM5*	rs136728112	47	62	75.81	intron

### Functional analysis of ASE genes

To understand the biological processes underpinning production traits that could be affected by genes showing ASE, we selected only genes showing at least five samples with ASE. The list of 396 SNPs showing ASE in at least five samples corresponded to 277 genes, from which DAVID software recognized 255 based on its bovine database. This gene list corresponded to 10 clusters of over-represented GO biological processes among which we can highlight two directly involved with muscle biology: muscle system and contraction; metabolic energy process. All the biological GO terms and genes are described in Supplementary Table S4.

In addition, we verified whether these ASE SNP-containing genes were associated with meat quality traits in previous studies in the same Nelore population^19–24^ by either GWAS or RNA-seq. We identified 192 ASE genes associated with a total of 15 production traits, of which 147 were associated uniquely from QTLs, 12 uniquely from RNA-Seq, and 33 from both approaches. *TMEM182* and *ALDH5A1* were previously associated with seven and six traits, respectively, including meat tenderness^21^.

Except for the *CASQ1* gene, the top 10 genes for ASE consistency described in Table 1 have also been related to at least one production trait (fat color^21^), while *CMYA5*, *TXNIP*, *PDE4DIP*, *HSPA1A*, and *DNAJC21* were also associated with ribeye area^21^. *SPARC* was additionally associated with intramuscular fat^20^, *CMYA5*, *HSPA1A*, and *PDLIM5* were associated with water holding capacity as well^21^, while *HSPA1A* was also associated with cooking loss^21^. Supplementary Table S5 describes all ASE genes associated with traits, the respective traits, and studies.

### Putative causes of ASE

eQTLs and CNVs are mechanisms that may affect not only the total expression of the genes but also the allelic expression. Therefore, eQTLs identified by Cesar *et al*. (2018) were used to find possible regulatory variants of allelic expression considering the genes used for the functional analysis, which showed at least five samples with ASE. This data integration indicated that 68 cis-eQTLs (Supplementary Table S6) affected the total expression of seven ASE genes, encompassing seven ASE SNPs. The *trans*-eQTL affected 22 genes. Two genes were affected by several *cis*-eQTLs, *ALDH5A1*and *RDH14*, affected by 38 and 21 *cis*-eQTLs, respectively. *RYR1* and *RFC5* genes were affected by one *cis-*eQTL, *SIRT5* and *ACOT13* were affected by two, and  *IFNGR2* by three.

We used the CNV regions detected by Silva *et al*.^25^ to find out which ASE genes could have the allele expression affected by CNVs. Fourteen ASE SNPs were in regions of CNVs, encompassing eleven genes, and two were in intergenic regions (Supplementary Table S7). The ASE gene *ARSG* had two SNPs in a CNV region that caused loss of copy number and was identified in 0.27% of the same Nelore base population from which we used a subsample^25^. The CNVR2393 that co-located with the ASE SNPs rs136775442 (*SLC9A3R2*) and rs133877387 (*MSRB1*) showed the higher percentage (4.84%) in this Nelore population^25^.

### Comparison with other muscle ASE study

We compared our findings with a recent published study that used 19 Limousine to find ASE genes in muscle tissue^18^. A total of 109 SNPs was common between both studies (Supplementary Table S8), which represented 13.29% of all ASE identified here, including the top 10 ASE genes *CMYA5*, *CASQ1*, *SPARC*, and *HSPA1A*.

## Discussion

Describing ASE is a useful approach for mapping genetic variants that affect gene regulation as well as for the identification of distortions from the expected additive model in predicting production traits. Despite that, limited information is available about ASE in the literature. For this reason, we mapped the ASE in the skeletal muscle transcriptome of Nelore steers, using the new bovine genome ARS-UCD1.2 as reference in cattle. Furthermore, we provide a functional analysis for the SNPs, their respective genes presenting ASE, and their putative role and function on essential biological processes related to skeletal muscle tissue.

We found extensive ASE distributed across the bovine transcriptome, as seen in previous studies in mouse^26^, humans^27^ and bovines^3,18^. We detected considerable ASE variation among individuals as well as among SNPs. However, we also identified SNPs showing total or high consistency of ASE patterns among individuals. The relevance of the patterns and mechanisms of allelic expression was evident in our study, considering that several genes playing critical biological functions in the muscle had unequal allelic expression and even monoallelic expression.

The allelic imbalance seems to be common since nearly 75% of the tested SNPs showed at least one individual with ASE. The positive correlation between number of tested samples and number of significant ASE SNPs was expected, as increasing samples should increase the number of SNPs meeting the test criteria, thus increasing the probability of identifying an ASE SNP. These findings are in accordance with other studies available in the literature. Crowley *et al*.^26^ found 89% of ASE for mouse using 96 samples, while Chamberlain *et al*.^3^ found similar results, ranging from 74 to 89%, but using one dairy cow and considering 18 tissues. However, when Chamberlain *et al*.^3^ analyzed only one tissue of this single animal, the semimembranosus leg muscle, the proportion decreased to 18%. The same authors observed considerable frequency increasing when the sample size was enlarged to 20 samples (for white blood cells and liver tissues). Additionally, we observed a great variation across individuals, as some had few SNPs showing ASE, with a minimum of 15%, and others reached 100% of tested SNPs with ASE. Other studies with livestock species showed results corroborating these findings. For instance, Oczkowicz *et al*.^28^, using 12 porcine brains found that 52% of the genes are subjected to ASE, while Ghazanfar *et al*.^29^ using only one sample, found 25%. Guillocheau *et al*.^18^, using 19 Limousine animals found 20% of the expressed genes having at least one SNP showing ASE.

We showed that genotype frequencies influenced the ability to observe ASE, since the variation of testable SNPs ranged from one to 104 among individuals depending mainly on the occurrence of heterozygosity. The ASE variation across individuals was described by the GTEX Consortium^30^ as well, who found higher correlation among different tissues within the same individual than among the same tissues originated from different individuals. The aggregation of our results and reports from literature reinforces the need for biological replicates in ASE studies, as already demonstrated by Lagarrigue *et al*.^8^. For the same reason, a word of caution is to be taken regarding ruling out the occurrence of ASE in the 265 SNPs that did not show ASE in any tested individual, since only three had more than ten tested individuals. Caution also should be taken when considering the frequency of ASE in the population as a criterion of relevance for consideration in genetic modeling, as this is a limited sample of the Nelore breed. In addition, some implications of the findings of this single-tissue study may not be generalized to a broader cross-section to other tissues, developmental stages, and species before a broader investigation is conducted.

The presence of an ASE pattern can be used as a filter in the search for imprinted genes, sparing effort and time by focusing only on genes with a difference of expression between parental alleles. For instance, here we presented 277 genes showing ASE in at least five samples, for which further studies could complement the knowledge of imprinted status in *Bos indicus* muscle tissue. From all those genes showing ASE pattern, only two were included in the public imprinted genes database Catalogue of Imprinted genes^31^. Since this database provides a collection of studies regarding parental origin available in the literature, this means that, from our list of 277 genes, only the genes *BLCAP*^32–34^ and *NNAT*^34–36^ were studied regarding the imprinted status heretofore. Studies about the parental origin of the expressed allele in bovine genes are relevant in particular when the intensity of selection is higher in a genus^37^, as is the case of beef cattle in which males are subjected to higher selective pressure. Here, we found monoallelic expression in 25.8% of tested SNPs, which could be potential candidates for imprinted status investigation in further studies. However, besides imprinting, monoallelic expression can also be a result of other mechanisms. Deng *et al*.^38^ reported monoallelic expression in 12–24% of the tested genes in mouse, independently of parental origin. Interestingly, our frequency of monoallelic SNPs was much higher than the 4.3% found by Chamberlain *et al*.^3^ in bovine semimembranosus muscle. This difference may be explained by the sample size, since those authors used a single cow, whereas we analyzed 190 individuals.

ASE is the base for several types of studies, as genomic imprinting, aseQTL, and reQTL, which can provide insights into the regulation of essential genes involved in organism maintenance. Functional analysis showed that the ASE genes were involved in biological processes underlying muscle development, structure, and function. Additionally, many of ASE genes identified here have been already associated with production traits in the same population. *CASQ1* gene, that showed a consistent allelic imbalance in 90% of 87 testable individuals, was clustered with biological processes directly involved with muscle structure (clusters 1 and 2) and with muscle contraction (cluster 4 and 5). Indeed, the product of this gene is part of the protein Calsequestrin, which is known to bind calcium and act in calcium storage, an essential mechanism for muscle contraction control through regulation of the calcium channel activity of *RYR1* gene^39^ in skeletal muscle. *RYR1* showed consistent ASE through the 27 individuals tested (81%). Besides the consistent allelic imbalance found in genes involved in the contraction mechanism, we found genes encoding proteins that act on skeletal muscle assembly, as *MYOM1*. This gene, with two ASE SNPs (81.5% and 68.42%) was highlighted in the functional analysis since its product is a protein (myomesin) component of sarcomeres^40^. Myomesin binds myosin proteins^41^ and together, they are essential to stabilize the sarcomere. Myosin proteins themselves might be affected by the ASE in *MYL1* gene, which encodes a myosin subunit that is an effector ATPase in muscle contraction^42^. The ASE pattern was observed in 63.26% of 98 testable individuals for the SNP rs109485588 on this gene This gene overlaps QTLs for two meat quality traits, water holding capacity and color parameters of intramuscular fat^21^. *MYL1* was also associated with increased oxidative damage in tender meat^43^. Altogether, functional analysis by over-representation of biological processes and the association with production traits reinforced the relevance of ASE genes for beef production phenotypes and the need for understanding their expression pattern.

As mentioned before, genes showing monoallelic expression are interesting for genomic imprinting studies. Particularly those genes associated with relevant animal production traits like *DHRS7B*, which had changes in its expression associated with fatty acid content in skeletal muscle^20^ and was related to energy process (cluster 6) in our functional annotation. This gene showed ASE in 84.21% of tested individual (16 out of 19) and, from those ASE individuals, 87.5% were monoallelic.

Another interesting gene showing high proportion of monoallelic expression was *ALDH5A1* (100% of ASE individuals), which was associated with energy metabolism and affects several production traits. In a GWAS study^21^, this gene was associated with water holding capacity, fat color parameters, and tenderness. In addition, 38 cis-eQTL were identified affecting the expression of this gene^21^.

The results regarding the conservation of ASE between samples for many genes, as is the case of genes showing higher consistency featured as higher number of ASE individuals and higher proportion of ASE/tested SNPs (top 10 genes – Table 1), suggest the existence of consistent *cis-* regulatory elements controlling the expression of these genes that were not found by the eQTL analysis carried by Cesar *et al*.^44^. In these cases, further studies adopting the aseQTL approach could be useful to detect the *cis*-element responsible for the allele expression imbalance.

Some of the ASE SNPs overlapped CNV regions previously identified in a larger population from which our samples were selected, suggesting the difference in number of copies could also be the cause of imbalance in allele-expression^25^.

Although other studies applied SNP calling from RNA-seq data as an alternative approach to detect ASE^3^, which may imply a more significant number of SNPs studied, we chose to use the variants present in the Illumina BovineHD BeadChip for two reasons. First, our methodology ensures lower errors in genotyping heterozygous samples as homozygous in case of monoallelic expression. Second, this array of SNPs is already available and used in genomic selection, thus understanding the expression patterns of those SNPs have potential to increase the accuracy of selection as the information of ASE SNPs was used to improve the accuracy of bird selection regarding susceptibility to the Marek disease virus infection^45,46^. Nevertheless, the first studies of genome-wide ASE in cattle were published only recently^3,18^, and despite the massive contribution of this work to the scientific community, there are still many open questions to be addressed.

We believe that our results will open a range of possibilities for further investigation. Epigenetic studies like parental origin effects and DNA methylation could add a layer of information about the regulation of ASE gene expression affecting traits of economic relevance. This epigenetic data may contribute to animal breeding by allowing the elucidation of causality with incomplete explanations regarding the heritability of complex traits. It could contribute to the reduction of the existing noise in the phenotype decomposition equation (infinitesimal model) to increase the accuracy of parameters estimation^47^.

Additional data from different tissues and developmental stages will be necessary to expand the knowledge of ASE status through the whole bovine organism. Furthermore, the identification of the presence of *cis*-regulatory elements in our results requires future investigation by an aseQTL approach to map the causal *cis*-acting variants.

Considering all data presented herein, it is evident that ASE is a common event through bovine transcriptome. It is present in genes involved in crucial biological processes as well as associated to QTLs. These results suggest that the deviation from the general model of biallelic expression may have substantial impacts over skeletal muscle assembling and function. In conclusion, this study achieved the goal of showing that biallelic expression may not be a general rule in *Bos indicus* skeletal muscle and that allelic expression affecting meat quality related genes could be considered as an extra layer of information to increase efficiency in animal breeding programs.

## Material and Methods

### Animals, DNA, and RNA

A total of 190 Nelore steers were raised in feedlots under identical nutrition and handling conditions until being slaughtered at an average age of 25 months. The steers are the offspring of 34 representative sires of the main commercialized Nelore genetic lines in Brazil. Details about animal production, raising and slaughtering were described previously^44^. All procedures involving steers were approved by the Institutional Animal Care and Use Committee Guidelines from Brazilian Agricultural Research Corporation – EMBRAPA (process number: Macroprograma 1, 01/2005) and sanctioned by the president Dr. Rui Machado to ensure compliance with international guidelines for animal welfare.

For sires, DNA was extracted from frozen semen by a standard phenol-chloroform method^48^. For steers, DNA was extracted from blood samples by a salting out method for which the details were published elsewhere^44^. DNA concentration was measured by spectrophotometry in a NanoDrop^®^ equipment, while quality was measured by the ratio of the optical absorbance 260/280, followed by integrity inspection by agarose gel electrophoresis.

The *Longissimus thoracis* muscle samples from 190 Nelore steers were immediately snap frozen in liquid nitrogen after collection during the slaughter and kept at −80 °C until RNA extraction. Total RNA was extracted from 100 mg of *Longissimus thoracis* muscle using TRIzol^®^ (Life Technologies, Carlsbad, CA, USA). RNA quality was analyzed by Bioanalyzer 2100^®^ (Agilent, Santa Clara, CA, USA), as described by Cesar *et al*.^44^.

### Genotyping

Genotyping using Illumina BovineHD BeadChip (Illumina Inc, San Diego, CA, USA), was carried out as previously described^49^ at the Bovine Functional Genomics Laboratory USDA - ARS (Beltsville, MD, USA), and at Functional Genomic Center - ESALQ, (Piracicaba, SP, Brazil). Quality control was performed using PLINK 1.9 software. Only autosomal SNPs corresponding to ARS1.2 (ftp://ftp.ensembl.org/pub/release-97/fasta/bos_taurus/dna/Bos_taurus.ARS-UCD1.2.dna.toplevel.fa.gz) genome coordinates were considered for analyses. Quality control filtering removed SNPs with a minor allele frequency <5% and a call rate <95% per SNP and per sample. In addition, we removed SNPs with a Hardy-Weinberg test <0.0001. Lack of population structuring was shown in a previous work^49^. The final dataset included 429,513 SNPs and 190 cattle. The linkage phase was determined using BEAGLE 4 software^50^.

### RNA Sequencing (RNA-seq)

Library preparation and sequencing were performed at the Functional Genomic Center - ESALQ (Piracicaba, SP, Brazil), as described by Cesar *et al*.^44^. TruSeq RNA Sample Preparation Kit (Illumina, San Diego, CA, USA) were used with 2 *µ*g of total RNA to assemble the library, following manufacturer’s protocol. The average size of the libraries was determined using Bioanalyzer 2100^®^ (Agilent, Santa Clara, CA, USA) and the quantification was performed by quantitative PCR using the KAPA Library Quantification kit (KAPA Biosystems, Foster City, CA, USA). Clustering and sequencing were carried out with Illumina HiSeq. 2500^®^ (Illumina, San Diego, CA, USA). Quality control was performed by Cesar *et al*.^44^. Briefly, Sequencing adapters and low complexity reads were removed with SeqyClean (https://github.com/ibest/seqyclean) and quality control was performed with FASTQC version 0.10.1 software (https://www.bioinformatics.babraham.ac.uk/projects/fastqc/).

### ASE analysis

ALEA software^51^ was employed to build a diploid genome for each animal using the bovine reference genome (*Bos taurus* ARS1.2 ftp://ftp.ensembl.org/pub/release-97/fasta/bos_taurus/dna/Bos_taurus.ARS-UCD1.2.dna.toplevel.fa.gz) and individual genotypes from Illumina BovineHD BeadChip. For this, we used the BEAGLE output file containing all the phased genotypes as input to ALEA to reconstruct the haplotypic regions based on the reference genome and to build two *in silico* genomes for each animal. Afterward, these genomes were concatenated in a single diploid genome for each animal. ALEA was executed accordingly to the manual guide (https://github.com/hyounesy/ALEA#quick-reference), except for the phaseVCF step for which we used BEAGLE^50^, as described before. For each individual, ALEA software use the phased haplotype to construct the diploid genome. The reads were then mapped to the diploid genome considering only perfect matches *ie*. every base pair must be exactly identical to the reference genome. Therefore, it is possible to identify reads that match a given allele and not other. Those SNPs showing less than 20 perfectly mapped reads^52^ were filtered out.

A binomial test was adopted to evaluate whether the read count was significantly different between both alleles of a specific SNP for each individual. The null hypothesis was that half of the reads mapped to one allele and half to the other. We applied a false discovery rate test (FDR) using Benjamini-Hochberg method (FDR < 0.05) for multiple test correction.

### SNPs and gene annotation

Annotation of ASE genes and SNPs was performed *in silico* by two approaches. First, we used the Variant Effect Predictor (VEP)^53^ with *Bos taurus* ARS-UCD1.2 as genome reference, and Ensembl for transcript database. The default parameters were used, except the Upstream/Downstream distance (bp) that was set as zero because only variants in transcribed regions were considered.

### Functional analysis

To predict the function of ASE genes and the biological processes affected by allele-expression patterns, we used Functional Annotation Clustering tool from DAVID version 6.8 (Huang *et al*., 2009). We used the Gene Ontology terms Biological Process (BP) FAT, and *Bos taurus* as background, using default parameters, except for the enrichment threshold (EASE) that we set as 0.05.

### Integration of ASE genes with previous studies

We performed an integration of ASE genes with results from previous studies in a larger population that comprised the animals used here. We checked which of the ASE genes were listed as associated with traits, either for being in a QTL window of a GWAS or being differentially expressed in RNA-Seq of contrasting groups for the given phenotype. The GWAS traits analyzed were intramuscular fat deposition and composition^19^, meat quality and yield traits^21^, and feed efficiency-related traits^23^. Traits associated through differential expression analysis were fatty acid content in skeletal muscle^20^, meat tenderness^54^, residual feed intake^22^, and muscle iron content^24^.

In addition, we checked whether an ASE gene was affected by an eQTL, described by Cesar *et al*.^44^, by comparing the list of eQTLs’ target genes with our ASE genes. Similarly, to check wether a CNV could be the cause of ASE, we used the list of CNV regions described by Silva *et al*.^25^ in the same Nelore population from which our samples were derived. For these comparisons, the original genome annotation assembly (UMD_3.1) was converted for the ARS-UCD1.2 in the UCSC Lift Genome Annotation tool (https://genome.ucsc.edu/cgi-bin/hgLiftOver). Finally, we compared the list of ASE SNPs (rs IDs) identified by Guillocheau *et al*.^18^ with our ASE SNPs.

## Supplementary information

Supplementary Dataset.

## Data Availability

The dataset supporting the conclusions of this article is available in the European Nucleotide Archive (ENA) repository (EMBL-EBI), under accession PRJEB13188, PRJEB10898, and PRJEB19421 (https://www.ebi.ac.uk/ena/submit/sra/).
